# Perceived stress, coping strategies, symptoms severity and function status among carpal tunnel syndrome patients: a nurse-led correlational Study

**DOI:** 10.1186/s12912-024-01761-y

**Published:** 2024-02-01

**Authors:** Maha Gamal Ramadan Asal, Mohamed Hussein Ramadan Atta, Sally Mohammed Farghaly Abdelaliem, Ahmed Abdelwahab Ibrahim El-Sayed, Hoda Abdou Abd El-Monem El-Deeb

**Affiliations:** 1 Medical Surgical Nursing Department, Faculty of Nursing, Alexandria University, 9 Edmond Vermont Street - Smouha, Alexandria, Egypt; 2 Psychiatric and mental health nursing Department, Faculty of Nursing, Alexandria University, 9 Edmond Vermont Street - Smouha, Alexandria, Egypt; 3 Nursing Administration Department, Faculty of Nursing, Alexandria University, 9 Edmond Vermont Street - Smouha, Alexandria, Egypt

**Keywords:** Perceived stress, Coping strategies, Symptoms severity, Function status, Carpal tunnel syndrome

## Abstract

**Background:**

Carpal tunnel syndrome (CTS) is a prevalent condition characterized by hand pain, tingling, and numbness. The severity of symptoms and functional status in CTS patients may be influenced by perceived stress and how individuals cope with it. However, scarce knowledge exists about the role of coping strategies as moderators in this relationship. Unfolding the role of perceived stress and coping strategies for CTS management will help the nurse to provide comprehensive and tailored nursing care. This will ultimately improve patient comfort, functionality, and quality of life.

**Purposes:**

This study aimed to examine the role of coping strategies (adaptive and maladaptive) in the relationship between perceived stress and both symptoms severity and function status among those patients.

**Method:**

We employed a multisite, correlational study design with moderation analysis. The study included 215 patients with CTS from neurosurgery outpatient clinics at three hospitals in Egypt. After obtaining their consent to participate, eligible participants completed anonymous, self-reported measures of perceived stress, the brief COPE inventory, and the Boston Carpal Tunnel Questionnaire. Demographic and biomedical data were also collected. The questionnaire took about 20 min to be completed. The data was collected over six months, starting in February 2023.

**Results:**

The results showed that perceived stress, adaptive coping, and maladaptive coping were significant predictors of symptoms severity and functional status. Adaptive coping moderated the relationships between perceived stress and both symptoms severity and function status, while maladaptive coping did not. The interaction between perceived stress and adaptive coping explained a moderate effect on symptoms severity and function status after controlling for the main effects and the covariates.

**Conclusion:**

This study explored the relationship between perceived stress, coping strategies, and outcomes in patients with CTS. The results indicate that nurses play a vital role in assessing and assisting patients to adopt effective coping strategies to manage perceived stress and alleviate symptoms and functional impairment. Moreover, the findings support the need for psychological interventions that address both perceived stress and coping strategies as a way to enhance the functioning status and quality of life of patients with CTS.

## Introduction

Carpal tunnel syndrome (CTS) is the most common peripheral nerve entrapment syndrome, affecting millions of people around the world. CTS accounts for about 90% of all neuropathy conditions [1]. Its prevalence is quite significant, with studies estimating that up to 10% of the population may be affected and 50 per 1000 persons in developed countries. The mean annual crude incidence of CTS was found to be 329 cases per 100,000, and the standardized incidence was 276. The condition is more commonly diagnosed in middle-aged individuals, but it can occur at any age. Older people between the ages of forty and sixty are a particularly vulnerable group [2]. The risk and prevalence of CTs are doubled in obese people. In addition, women are more likely than men to experience CTS with a 3:1 ratio [3]. The risk of CTS is higher for men between the ages of 75 and 84, while it is more common for women between the ages of 45 and 54 [2].

CTS results from compression or irritation of the median nerve, which runs from the forearm to the hand and passes through the carpal tunnel in the wrist. CTS causes pain, numbness, tingling, and weakness in the wrist, the hand, and fingers, especially the thumb, index, middle, and ring fingers. The symptoms vary in severity and worsen at night or during activities that involve repetitive or forceful hand movements or gripping [4]. CTS can impair fine motor skills, such as writing, typing, or gripping objects. The severity of CTS determines how much it affects hand function and coordination. Chronic damage results as the disease worsens over time, causing muscular wasting, loss of grip strength, trouble with work activities, and social interaction. As a result, patients’ everyday activities are impacted, and their quality of life is diminished [5].

Several factors can contribute to the development of CTS. These include the anatomy of the wrist, health problems, and repetitive hand motions. Other factors that may increase the risk of developing carpal tunnel syndrome include being obese, pregnancy, diabetes, high blood pressure, and arthritis [6]. Lifestyle factors such as smoking, high salt intake, a sedentary lifestyle, and a high body mass index (BMI) may also increase the risk of developing CTS. The anatomy of the wrist can play a role in the development of CTS. A wrist fracture can narrow the carpal tunnel and irritate the median nerve, as can the swelling and inflammation caused by rheumatoid arthritis. Additionally, people who have smaller carpal tunnels or who have a bone arrangement that impinges on the carpal tunnel may be more susceptible to developing CTS [7].

Perceived stress and CTS are linked together. The reason is because emotional pain, stress, and anxiety change the way the body functions. When the body is under stress, it produces hormones that can cause inflammation and swelling in the body. This inflammation can put pressure on the median nerve, leading to the development of CTS [8]. Meanwhile, stress can definitely exacerbate CTS as it produces an indirect effect on the carpal tunnel space and median nerve deep inside. Thus, emotional stress causes a domino effect that worsens CTS. In addition, patients’ fear of losing their jobs makes them withhold symptoms, and the burden of maintaining functionality at work despite pain and discomfort is a powerful stressor for CTS patients. Furthermore, CTS and its associated functional impairments can create additional stressors in one’s life, such as personal losses and social isolation. These stressors can lead to heightened psychological distress, which can increase pain perception and stress responses and impair functional recovery [6].

Functional status refers to the ability of an individual to perform daily activities. CTS can have a significant impact on functional status, particularly if the condition is severe. In some cases, individuals with severe CTS may be unable to perform simple tasks such as holding a cup or opening a jar. The severity of functional impairment varies from individual to individual, but it is common for patients to struggle with tasks that were once effortless [1]. It is important to note that functional impartment in CTS produces a high level of stress among patients due to the impact of the condition on their daily lives. Dealing with chronic pain and functional limitations can lead to frustration, anxiety, and even depression. The inability to perform tasks that were once routine and the fear of losing independence can take a toll on a patient’s mental well-being [9]. Additionally, the uncertainty of future treatment options or the possibility of permanent disability could enhance the perceived stress levels among patients, which is a catastrophic issue in the course of CTS [9].

Coping of CTS patients with the resultant functional limitations as well as efficient stress management are the golden keys toward reducing the chronic magnitude and severity of CTS. Also, the ways the patient uses to cope could shape the bad prognosis and permanent disability that may be encountered. Coping strategies could be adaptive or maladaptive based on how the patient perceives his illness and is aware of possible outcomes [10]. Meanwhile, stress control through proper coping could be helpful in maintaining a healthy level toward symptoms alleviation. Furthermore, adaptive coping could reduce stress levels among CTS patients, which in turn would reduce the severity of symptoms and improve their functional status. Conversely, maladaptive coping produces further stress, which definitely exacerbates symptoms and induces functional limitation on a larger scale [11].

Adaptive coping strategies are effective in reducing stress and improving psychological and physical well-being. These strategies include seeking support from friends, family, or support groups; alternating job functions; avoiding repetitive motions; scheduled rest and breaks; heat and cold compresses; problem-solving techniques such as ergonomic adjustments or wrist splints; relaxation techniques such as deep breathing or mindfulness; and maintaining a positive mindset [5, 12]. Adaptive coping strategies help manage and alleviate CTS symptoms. On the other hand, maladaptive coping strategies, such as denial of symptoms, abuse of over-the-counter medications, avoiding activities that cause pain, or self-isolating from others, can lead to muscle weakness and deconditioning, which further limit hand function and delay healing. They can also create a sense of loss, frustration, helplessness, and isolation. These negative emotions can increase pain perception, stress responses, and functional recovery [13].

Nurses have an important role to play in the prevention and management of CTS. By identifying patients who are at risk, providing education on how to prevent the condition, and working with other healthcare professionals to develop treatment plans, nurses can help improve outcomes for patients with CTS [14]. Nurses play an important role in providing interventions that decrease CTS symptoms and increase functional status. The nurse should instruct the patient on practicing hand exercises that can minimize CTS symptoms and apply mild heat to the hands before practicing them. The nurse should also advise them to use the night wrist splint to support and immobilize the wrist at night. Moreover, nurses can educate CTS patients about the appropriate adaptive coping mechanisms and stress control measures based on their conditions [15].

### The significance of the study and the research gap

Contemporary nursing and medical literature places much emphasis on the predisposing factors and treatment modalities of CTS [1, 14]. Moreover, recent literature argues that psychological factors play a more pivotal role than physical factors in controlling the severity of symptoms and upgrading the functional status of patients with CTS [16]. However, there is a dearth of studies examining the role of psychological factors in the treatment course of CTS patients. Anxiety, pain intensity, self-efficacy, and depression are the most studied psychological factors and have gained momentum in the literature in terms of their role in shaping the severity and functional status of patients with CTS [8]. For instance, a systematic review by Sheikhzadeh et al. (2021) revealed that modifiable psychological factors, such as anxiety, depression, coping strategies, and self-efficacy, affected the outcomes of patients with musculoskeletal disorders, however managed by conservative or surgical treatment. The review suggested that interventions targeting modifiable psychological factors could improve patients’ outcomes and satisfaction [17].

In addition, Daliri et al. (2022) compared the correlation of depression, pain intensity, and anxiety with limb disability in patients with CTS. Their results revealed that anxiety was a significant predictor of disability and pain in both groups, which suggested that psychological factors are important determinants of self-reported symptoms severity and functional status in patients with CTS [18]. Furthermore, Harris-Adamson et al. (2022) found that personal, psychosocial, biomechanical, and job factors prophesied CTS-related disability. Their study concluded that prevention of severe disability needs acting on both psychological and biomechanical work stressors [19].

In this essence, perceived stress and coping mechanisms among patients with CTS received little consideration in contemporary literature; however, recent scholars argued that stress perception and the ways the patient with CTS uses to cope with stress and disability could worsen or improve both the symptoms of CTS and functional status [20, 21]. Although the relationship between perceived stress, coping mechanisms, symptoms severity, and functional status among patients with CTS seems original at first glance, the exact role of perceived stress and coping toward symptoms severity and functional status among those patients has not been adequately explained in the literature. To address this research gap, our study aims to examine the meticulous role of perceived stress and coping strategies toward symptoms severity and functional status among CTS patients.

Understanding how psychological factors like perceived stress and coping strategies affect the quality of life of patients with CTS in terms of functional abilities and severity of symptoms is of paramount importance for nursing practice. This is because nurses can use our findings to develop tailored nursing care plans that consider both physical and psychological variables affecting CTS patients. Also, nurses can use our results to provide evidence-based nursing care using a holistic patient-centered approach, which ultimately improves the quality of life and controls the chronic burden of patients with CTS. Our study has three objectives: first, to assess the levels of perceived stress, coping strategies, symptoms severity, and function status among CTS patients; second, to analyze the association between perceived stress, coping strategies, and CTS outcomes (symptoms severity and function status); and third, to examine the moderating role of coping strategies on the relationship between perceived stress and CTS outcomes. Based on these objectives, the following hypotheses were proposed:

#### H_1_

Perceived stress will be significantly and positively associated with the severity of symptoms.

#### H_2_

Perceived stress will be significantly and positively associated with functional status.

#### H_3_

Adaptive coping will moderate the associations between perceived stress and the severity of symptoms. Such that the positive association between perceived stress and the severity of symptoms will be weaker for patients with high adaptive coping scores than those with low scores.

#### H_4_

Adaptive coping will moderate the associations between perceived stress and functional status. Such that the positive association between perceived stress and functional status will be weaker for patients with high adaptive coping scores than those with low scores.

#### H_5_

Maladaptive coping will moderate the associations between perceived stress and symptoms severity. Such that the positive association between perceived stress and symptoms severity will be stronger for patients with high maladaptive coping scores than those with low scores.

#### H_6_

Maladaptive coping will moderate the associations between perceived stress and functional status. Such that the positive association between perceived stress and function status will be stronger for patients with high maladaptive coping scores than those with low scores.

**Fig. 1:**
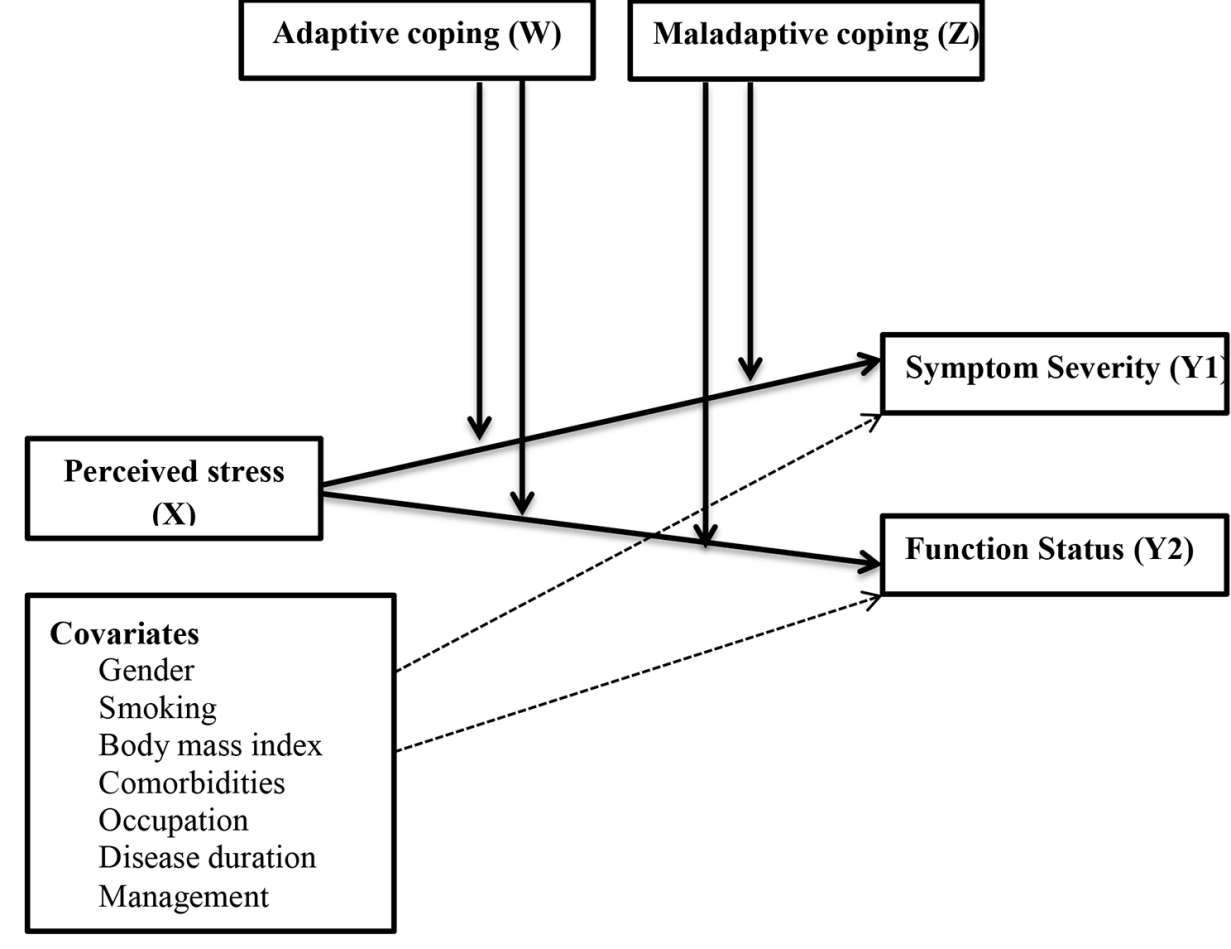
Conceptual model of the study

Figure 1 shows a conceptual model of the study illustrating the proposed relationship between perceived stress, coping strategies, symptoms severity, and function status among patients with CTS.

## Materials & methods

### Design and setting

We employed a multisite, correlational study design with moderation analysis. To fulfillthe study aim and test the proposed hypotheses, the correlational design was employed to identify the strength and direction of the relationship between the study variables. In addition to examining whether the relationship between perceived stress and outcomes in patients with CTS depends on the level of coping strategies, we employed a moderation model using multiple linear regression.

This study was conducted at the outpatient clinics of three hospitals in Alexandria, Egypt. These hospitals are the Main University Hospital, Alhadra University Hospital, and Gamal Abdel-Naser Health Insurance Hospital. These settings were selected because of their inclusion of specialized neurosurgery clinics that provide diagnostic, therapeutic, referral, and follow-up services for patients with peripheral nerve affections, serving Alexandria and the surrounding governorates. The study adheres to the STROBE reporting guidelines.

### Participants and sampling

Using the G*Power (3.1.9.7) program, we estimated that about 200 patients were required to attain 0.8 power with a type I error of 0.05, an effect size of 0.05, a total of 12 predictors, and two tested predictors for multiple linear regression analysis. The sample was increased to 225 to account for attrition bias.

The study participants included a convenience sample of 225 patients diagnosed with CTS of the median nerve who attended the above setting from February 2023 to the end of May 2023. The inclusion criteria were patients over 18 years old, literate, with an established diagnosis of CTS by a combination of clinical features, physical examination, and, according to nerve conduction studies findings, who attended outpatient clinics of the above-mentioned hospitals. Patients who had other causes of peripheral neuropathy, had CTS surgery within the last year, or were complaining of other neuropsychiatric diseases or cognitive limitations as reported by the patients or documented on the patients’ medical records were excluded.

### Study instruments

In this study, we used three valid Arabic-version instruments: the perceived stress scale-14, the brief COPE inventory, and the Boston carpal tunnel questionnaire to assess the study variables. In addition, the socio-demographic (e.g., age, gender, marital status, etc.) and clinical data (e.g., presence of comorbidities, duration of illness, BMI (Kg/m^2^), treatment modalities, etc.) of the study participants were also collected.

### Perceived stress scale (PSS-14)

The perceived stress was assessed using the Perceived Stress Scale (PSS-14), developed by Cohen et al. (1983) [22]. The PSS-14 has seven positive and seven negative items. The PSS-14 measures perceived stress using a 5-point Likert scale, where 0 = ‘never’ and 4 = ‘very often’. The total score of PSS is the sum of the negative items and the reverse-scored positive items, which can vary from 0 to 56. A higher score reflects more perceived stress [23]. The scale developer reported its convergent validity as measured by the associations with depressive (*n* = 332, *r* = 0.76) and physical (*n* = 64, *r* = 0.70) symptom scales. The Cronbach’s alpha coefficient ranged from 0.84 (*n* = 332) to 0.86 (*n* = 64), indicating a high internal consistency [22]. The PSS-14 has been applied in many contexts and has been demonstrated to be associated with several physiological and psychological indicators of stress [24]. The PSS has been translated into many languages, and its validity and reliability have been confirmed in Swedish [25], Japanese [24], Mexico [26], and Chinese [27].

An Arabic version of the PSS-14 was utilized in our study; the scale showed adequate validity and reliability with Cronbach’s α coefficient of 0.80, and the test-retest reliability had an intra-correlation coefficient of 0.90. Thus, the version is an appropriate tool to measure perceived stress in Arabic people [28]. The Cronbach’s α coefficient of the scale in our study was 0.906, which indicates a high internal consistency of the scale.

### Brief COPE inventory (brief COPE)

The Brief COPE Inventory (Brief COPE) is a 28-item scale that measures the frequency of using 14 different coping strategies in response to stressors [29]. The coping strategies are divided into two categories: adaptive and maladaptive. Adaptive coping includes nine strategies, such as planning, active coping, acceptance, positive reframing, emotional support, and humor. Maladaptive coping includes six strategies, such as denial, self-distraction, self-blame, substance use, and venting. The Brief COPE uses a four-point Likert scale, from 1 (not at all) to 4 (a lot), to rate each item. The scores for adaptive coping and maladaptive coping can range from 9 to 64 and from 6 to 48, respectively, with higher scores meaning more frequent use of the coping type. The brief COPE exhibited adequate psychometric properties, as reported by the developing authors [29]. In addition, the scale has good reliability and validity in different health-related research settings [5]. The Brief-COPE is translated in a number of languages, such as Urdu [30], Spanish [31], French [32], Brazilian-Portuguese [33], and Arabic [34]. The Arabic version of the brief COPE inventory was used in our study. The Arabic version exhibited good convergent validity and reliability, with Cronbach’s α coefficient ranging from 0.75 to 0.84 and test-retest reliability having an intra-correlation coefficient of 0.80 [34]. In our study, the Cronbach’s α coefficient of the Brief COPE inventory was 0.835 for the total scale, 0.901 for adaptive coping, and 0.898 for maladaptive coping, indicating high scale reliability and internal consistency.

### Boston carpal tunnel questionnaire (BCTQ)

The Boston Carpal Tunnel Questionnaire (BCTQ) is a patient-reported outcome instrument that assesses the symptoms severity and functional status of CTS patients [35]. It comprises two subscales: the Symptom Severity Scale (SSS), which has 11 questions, and the Functional Status Scale (FSS), which contains 8 items. Respondents rate the items for their degree of difficulty on a 1–5-point Likert scale. The final score is generated by summing the individual scores divided by the number of items. The SSS score ranges from 11 to 55 points, and the FSS score ranges from 8 to 40, with a higher score indicating greater disability [36]. BCTQ has been translated into several languages and has been found to have high levels of reliability and validity in different cultures [37–42]. The Arabic version of the BCTQ in the work of Alanazy et al. (2019) was used in our study. In the Alanazy et al. study, the Cronbach’s α was 0.91 for the total BCTQ score, 0.88 for the SSS, and 0.87 for the FSS [42]. In our study, the BCTQ had a high reliability of 0.927 for the total scale, 0.906 for the SSS, and 0.803 for the FSS.

In order to ensure the cultural relevance and appropriateness of the tools and the study rigor, the adopted Arabic versions were examined for content validity by five expert panels of medical-surgical, psychiatric and mental health, and neurosurgery specialists from academic settings. The tools were then pilot-tested on 30 patients to evaluate their clarity and readability. No modifications were suggested based on panel opinion and the pilot. These patients were not included in the study sample.

### Data collection

Data were collected over six months, starting in February 2023. A total of 270 patients were assessed for eligibility; 52 patients did not meet the recruitment criteria. As a result, 255 patients who met the inclusion criteria were invited to participate in the study after explaining the aim and nature of the study, ensuring their ability to refuse participation or withdraw at any time without any negative effects, and confirming that their information was confidential and that non-participation did not affect the medical services provided. The entire group of invited participants agreed to take part in the study; thus, an anonymous questionnaire was hand delivered to those patients. The questionnaires were distributed after the participants’ clinic appointment to avoid any interruption in completing the questionnaire and to ensure participants interest after getting their service at the clinic appointment. The questionnaire was completed by patients’ self-reports and took an average of 20 min to complete. A researcher was available at the clinic during the data collection to explain any unclear or ambiguous terms.

### Statistical analysis

**Fig. 2:**
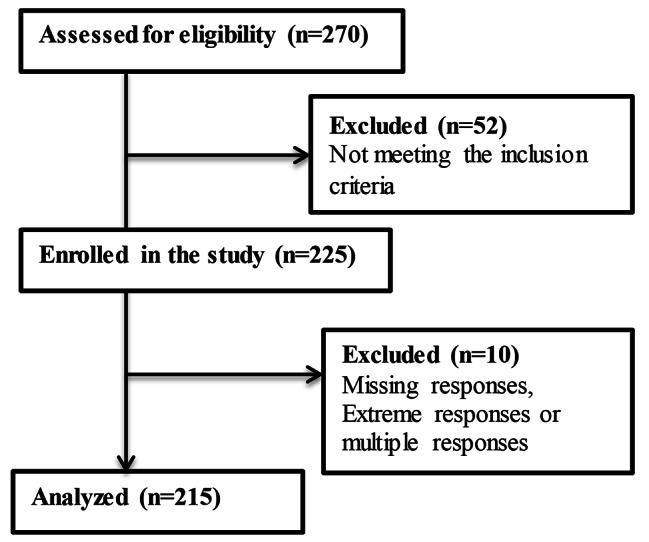
Flow diagram of the study

The IBM SPSS software package, version 25.0, was used to analyze the data. Data cleansing was carried out, and 10 respondents were excluded from the analysis due to missing, multiple, or extreme responses to address respondent bias; thus, 215 participants were analyzed (see Fig. 2). Descriptive analysis (mean, standard deviation, frequency, and percentage) was used to summarize and present the features of the demographic and biomedical data of the study participants and variables. The correlation analysis between the study variables, including perceived stress, adaptive coping, maladaptive coping, symptoms severity, and function status, was done using Pearson’s coefficient ® to measure the strength and direction of the linear relationship between each pair of variables. The *p*-value was used to test the significance of the correlation coefficient, with a threshold of 0.05.

To examine the moderating role of coping strategies (adaptive and maladaptive) on the relationship between perceived stress and symptoms severity, we performed multiple linear regressions using the SPSS PROCESS-macro (version 4.2) plugin. The PROCESS is widely used in the social, business, and health sciences for estimating two- and three-way interactions in moderation models along with simple slopes and conditional effects with a single or multiple moderators. The PROCESS macro is a free, easy, and useful method. In addition, the analysis provides the necessary data to draw the graphs where the effect can be observed. For this reason, the PROCESS v4.2 macro was used in the analysis [43].

We used the moderated moderation model No. 2 with symptoms severity as the dependent variable (Y) in model A and the function status in model B as the outcome variable, perceived stress (X) as the focal predictor, adaptive coping (W) and maladaptive coping (Z) as the moderators, and several demographic and clinical variables as the covariates. The continuous predictors were mean-centered to decrease the potential multicollinearity. Initially, we estimated the models fit for linear regression. The model fit parameters were good for both models, as estimated by the ANOVA (F) and the value of R^2^ [44]. For Model A, (F (18,126) = 28.48, *p* < 0.001, R^2^ = 0.723). For Model B, (F (18,126) = 27.64, *p* < 0.001). In addition, the model fit parameters improved after the addition of the interaction terms in both models: model A (F (2,196) = 7.29, *p* < 0.001, R^2^ change = 0.0206) and model B (F (2,196) = 6.037, R^2^ change = 0.0174, *p* = 0.002). These results indicate that the overall models were statistically significant, verifying that the data was suitable for regression and moderation analysis. Thus, our proposed models were supported for linear regression analysis.

To generate the conditional effect of the moderators, which is the effects of perceived stress on a CTS outcome at different levels of coping strategies. We decided on the levels of adaptive and maladaptive coping; we used one standard deviation below the mean as a low level, one standard deviation above the mean as an average level, and one standard deviation above the mean as a high level. Interaction plots are used to visualize these conditional effects using SPSS syntax. The study determined the significance of the effects based on 5000 random samples using the 95% bootstrap confidence interval (CI). There is a significant effect if the 95% CI does not include 0 [43]. In addition, the effect size (Cohen’s ƒ^2^) of the interaction terms was calculated as follows ${f}^{2}=\frac{{R}^{2}}{1-{R}^{2}}$ [45]. For interpreting the interaction terms impact, ƒ^2^ values of 0.005, 0.01, and 0.025 represent small, medium, and large effect sizes, respectively [46].

## Results

### Demographics and biomedical data of the study participants

Table 1 displays the study participants’ distribution according to demographic and biomedical data. The study included 215 patients with CTS, with a mean age of 45.72 ± 13.16 years. The majority of the participants were female (76.7%), hand workers (64.7%), married (84.7%), and had a basic education (45.6%). The mean BMI of the participants was 27.91 ± 3.26 kg/m2, indicating that most of them were overweight (53.5%) or obese (27%). More than half of the participants were current smokers (52.6%) and had comorbidities (60.5%). The mean disease duration was 3.91 ± 3.26 years, with most of the participants having the disease for 1–5 years (80.9%). The most common management methods used by the participants were hand bandages or wrist braces (85.1%), medicine (60%), and physiotherapy (29.3%). Only 18.1% of the participants had surgery for their condition.

**Table 1:** Distribution of the study participants according to demographic and biomedical data (*n* = 215)

	No	%
**Age (year)**		
20–40	93.0	43.3
41–60	79.0	36.7
61–74	43.0	20.0
**Mean ± SD**	**45.72 ± 13.16**	
**Gender**		
Female	165.0	76.7
Male	50.0	23.3
**Occupation**		
Hand work	139.0	64.7
Employed	55.0	25.6
Did not work or retired	21.0	9.8
**Educational level**		
Basic education#	98.0	45.6
Secondary education	88.0	40.9
Higher education	29.0	13.5
**Marital status**		
Single	33.0	15.3
Married	182.0	84.7
**Body mass index(Kg/m** ^**2**^ **)**		
Normal	42.0	19.5
Over-weight	115.0	53.5
Obese	58.0	27.0
**Mean ± SD**	**27.91 ± 3.26**	
**Smoking status**		
Current smoker	113.0	52.6
Non-smoker or Quitter	102.0	47.4
**Comorbidities**		
Yes	130.0	60.5
No	85.0	39.5
**Disease duration (year)**		
1–5	174.0	80.9
6–10	36.0	16.7
11–14	3.0	2.3
**Mean ± SD**	**3.91 ± 3.26**	
**Managemen**t*		
Use of medicine	129.0	60.0
Use of hand bandage or wrist brace	183.0	85.1
Traditional Chinese medicine	13.0	6.0
Physiotherapy	63.0	29.3
Surgery	39.0	18.1

*Multiple responses were allowed, # includes pre- primary, primary and preparatory levels

### Descriptive statistics and correlations among the study variables

Table 2 presents the descriptive statistics of the study variables. It shows that the average level of perceived stress in the sample was 26.06 ± 8.09. The average level of adaptive coping was 45.57 ± 8.19. The average level of maladaptive coping was 32.20 ± 6.93. The average level of symptoms severity was 31.48 ± 6.94. The average level of functional status in the sample was 23.57, with a variation of about 5 points around the mean.

Table 2 also shows the results of a correlation analysis between the study variables. The results indicate that all the correlations between the study variables were statistically significant at *p* ≤ 0.05. The correlation coefficients ranged from − 0.450 to 0.822, indicating varying degrees of positive and negative associations. The results show that perceived stress had a positive correlation with maladaptive coping (*r* = 0.268, *p* < 0.001), symptoms severity (*r* = 0.506, *p* < 0.001), and functional status (*r* = 0.619, *p* < 0.001), and a negative correlation with adaptive coping (*r* = -0.375, *p* < 0.001) This suggests that higher levels of perceived stress were associated with higher levels of maladaptive coping, symptoms severity, poor functional status, and lower levels of adaptive coping.

The results also show that maladaptive coping had a positive correlation with symptoms severity (*r* = 0.435, *p* < 0.001) and functional status (*r* = 0.43, *p* < 0.001), indicating that higher levels of maladaptive coping were associated with higher levels of symptoms severity and poor function status. In addition, adaptive coping had a significant negative correlation with maladaptive coping (*r* = -0.157, *p* = 0.022), symptoms severity (*r* = -0.450, *p* < 0.001), and functional status (*r* = -0.373, *p* < 0.001), indicating that higher levels of adaptive coping were associated with lower levels of maladaptive coping, and better CTS outcome. The table also shows that symptoms severity had a significant positive correlation with functional status (*r* = 0.822, *p* < 0.001), indicating that higher levels of symptoms severity were associated with greater impairment in functional status.

**Table 2:** Descriptive statistics and correlation between the study variables (*n* = 215)

		Perceived stress	Adaptive coping	Maladaptive Coping	Symptoms severity	Functional status
Perceived stress	R	-				
	P	-				
Adaptive coping	R	− 0.375^*^				
	P	< 0.001^*^				
Maladaptive coping	R	0.268^*^	− 0.157^*^			
	P	< 0.001^*^	0.022^*^			
Symptoms severity	R	0.506^*^	− 0.450^*^	0.435^*^		
	P	< 0.001^*^	< 0.001^*^	< 0.001^*^		
Functional status	R	0.619^*^	− 0.373^*^	0.43^*^	0.822^*^	-
	P	< 0.001^*^	< 0.001^*^	< 0.001	< 0.001	-
Mean ± SD		26.06 ± 8.09	45.57 ± 8.19	32.20 ± 6.93	31.48 ± 6.94	23.57 ± 4.95

r: Pearson coefficient, *: Statistically significant at *p* ≤ 0.05

### Moderating role of coping strategies (adaptive and maladaptive) in the relationship between perceived stress and both symptoms severity and functional status among the study participants

Table 3 shows the results of two moderation analyses that examined the role of coping strategies (adaptive and maladaptive) in the relationship between perceived stress and symptoms severity (model A) and functional status (model B) among the study participants. For model A, the predictors in the model explained 72.3% of the variance in symptoms severity. Among the covariates, BMI (B = 0.295, *p* = 0.001) and the presence of comorbidities (B = 7.76, *p* < 0.001) were positively associated with symptoms severity, indicating that higher BMI and the presence of comorbidities were related to more severe symptoms. None of the other covariates had a significant effect on symptoms severity.

**Table 3:** Moderating role of coping strategies on the relationship between perceived stress and symptoms severity among the study participants (*n* = 215)

Variables	Model A (DV = Symptom Severity)						Model B (DV = Function Status)					
	B	se	T	p	95% CI		B	se	T	P	95% CI	
					LL	UL					LL	LL
**Constant**	27.85	1.28	21.61	< 001	25.31	30.39	20.59	0.928	22.17	< 0.001	18.76	22.42
**Covariates**												
Gender	− 0.655	0.70	− 0.936	0.35	-2.03	0.725	− 0.336	0.504	− 0.667	0.50	-1.33	0.658
Age	− 0.001	0.025	− 0.014	0.988	− 0.05	0.049	0.003	0.018	0.210	0.83	− 0.032	0.039
Smoking	− 0.193	0.564	− 0.343	0.732	-1.307	0.920	0.590	0.407	1.45	0.14	− 0.211	1.39
Body mass index(Kg/m^2^)	0.295	0.092	3.204	0.001	0.113	0.477	0.136	0.066	2.04	0.04	0.004	0.267
Comorbidities	7.76	0.687	11.29	< 0.001	6.40	9.11	4.81	0.495	9.71	< 0.001	3.83	5.78
Handwork occupation^£^	0.669	1.004	0.666	0.505	-1.31	2.64	0.390	0.723	0.539	0.59	-1.03	1.81
Employed^£^	0.421	1.109	0.38	0.704	-1.76	2.61	0.907	0.799	1.13	0.25	− 0.670	2.48
Disease duration	0.091	0.132	0.686	0.493	− 0.170	0.352	− 0.035	0.095	− 0.369	0.71	− 0.223	0.153
Use of medicine	− 0.035	0.567	− 0.062	0.949	-1.15	1.08	0.296	0.409	0.725	0.46	− 0.510	1.10
Use of hand bandage or wrist brace	-1.42	0.778	-1.82	0.069	-2.95	0.114	-1.34	0.560	-2.39	0.01	-2.45	− 0.239
Traditional Chinese medicine	1.06	1.14	0.935	0.35	-1.183	3.31	0.746	0.822	0.90	0.36	− 0.875	2.36
Physiotherapy	− 0.396	0.594	− 0.666	0.505	-1.56	0.775	0.547	0.428	1.27	0.20	− 0.297	1.39
Surgery	− 0.651	0.903	− 0.721	0.47	-2.43	1.12	0.122	0.650	0.188	0.85	-1.16	1.40
**Main effect**												
Perceived stress	0.148	0.038	3.86	< 0.001	0.072	0.223	0.218	0.027	7.90	< 0.001	0.164	0.273
Adaptive copping	− 0.091	0.038	-2.36	0.018	− 0.167	− 0.015	0.006	0.027	0.243	0.80	− 0.048	0.061
Maladaptive coping	0.130	0.042	3.08	0.0023	0.047	0.213	0.107	0.030	3.53	< 0.001	0.047	0.166
**Interaction effect**												
XW: Perceived stress× adaptive copping	− 0.014	0.004	-3.49	< 0.001	− 0.023	− 0.006	− 0.0095	0.003	-3.11	0.002	− 0.015	− 0.003
XZ: Perceived stress× maladaptive copping	0.002	0.005	0.456	0.64	− 0.008	0.013	0.0022	0.003	0.581	0.56	− 0.005	0.009
	R^2^ = 0.723,F(18,126) = 28.48 *,*p* < 0.001* XW: F(1,196) = 12.24, *p* < 0.001,R^2^ change = 0.0173 XZ: F(1,196) = 0.208, *p* = 0.64,R^2^ change = 0.001 BOTH: F(2,196) = 7.29, *p* < 0.001,R^2^ change = 0.0206						R^2^ = 0.717,F(18,126) = 27.64 ^*^,*p* < 0.001^*^ XW F(1,196) = 9.67, *p* = 0.002,R^2^ change = 0.0140 XZ:F(1,196) = 0.338, *p* = 0.056,R^2^ change = 0.001 BOTH: F(2,196) = 6.037, *p* = 0.0029,R^2^ change = 0.0174					

f, p: *f* and *p* values for the model, R^2^: Coefficient of determination, B: Unstandardized Coefficients, se: standard error, t: t-test of significance, LL: Lower limit, UL: Upper Limit, CI: confidence interval, ^£^: dummy coded variables as not work = 0*: Statistically significant at *p* ≤ 0.05

The results also show that the main effect of perceived stress was positive and significant (B = 0.148, *p* < 0.001), indicating that higher perceived stress was associated with higher symptoms severity. The main effect of adaptive coping was significant and negative (B= -0.091, *p* = 0.018), indicating that higher adaptive coping was associated with lower symptoms severity. The main effect of maladaptive coping was significant and positive (B = 0.130, *p* = 0.0023), indicating that higher maladaptive coping was associated with higher symptoms severity. These indicated that BMI, the presence of comorbidities, perceived stress, adaptive coping, and maladaptive coping were significant predictors of the severity of the symptoms among the study participants.

Regarding the moderating role of adaptive and maladaptive coping on model A, the results show that the interaction between perceived stress and adaptive coping was significant and negative (B = -0.014, *p* < 0.001). This means that it weakened the positive association between perceived stress and symptoms severity. In other words, people who used more adaptive coping strategies had lower symptoms severity when they experienced high levels of perceived stress.

This interaction explained a moderate proportion of variance in the symptoms severity after controlling for the main effects and the covariates (XW: F (1,196) = 12.24, R^2^ change = 0.0173, *p* < 0.001) with a medium effect size (ƒ^2 =^ 0.0174).

The interaction between perceived stress and maladaptive coping was not significant (B = 0.002, *p* = 0.64). The interaction explained a very small variance in the symptoms severity (XZ: F (1,196) = 0.208, R^2^ change = 0.001, *p* = 0.64) with a small effect size (ƒ^2^ = 0.001).

For model B, the predictors in the model explain 71.7% of the variance in the function status. Among the covariates, BMI (B = 0.14, *p* = 0.04), and the presence of comorbidities (B = 4 0.81, *p* < 0.001), were positively associated with function status, indicating that higher BMI and the presence of comorbidities were related to poor function status. The use of a hand bandage or wrist brace (B = -1.35, *p* = 0.02) was negatively associated with function status, indicating that the use of a wrist brace or hand bandage was related to better function status. None of the other covariates had a significant effect on the functional status.

The main effect of perceived stress was significant (B = 0.218, *p* < 0.001), indicating that higher levels of perceived stress were associated with lower levels of functional status. The main effect of adaptive coping was not significant (B = 0.006, *p* = 0.80), suggesting that adaptive coping did not have a direct impact on functional status. The main effect of maladaptive coping was significant (B = 0.107, *p* < 0.001), indicating that higher levels of maladaptive coping were associated with poor function status.

The interaction effect of perceived stress and adaptive coping was significant (B = -0.0095, *p* = 0.002), indicating that adaptive coping moderated the relationship between perceived stress and functional status. Specifically, the positive association between perceived stress and functional status was weaker for patients who used adaptive coping strategies. This interaction explained a moderate proportion of variance in the function status after controlling for the main effects and the covariates (XW: F (1,196) = 9.67, R^2^ change = 0.0140, *p* = 0.002) with a medium effect size (ƒ^2^ = 0.014).

The results also show that in model B, the interaction effect of perceived stress and maladaptive coping was not significant (B = 0.0022, *p* = 0.56), suggesting that maladaptive coping did not moderate the relationship between perceived stress and functional status. This interaction contributed to a small variance in the function status (XZ: F (1,196) = 0.338, R^2^ change = 0.001, *p* = 0.056) with a small effect size (ƒ^2^ = 0.001).

**Fig. 3:**
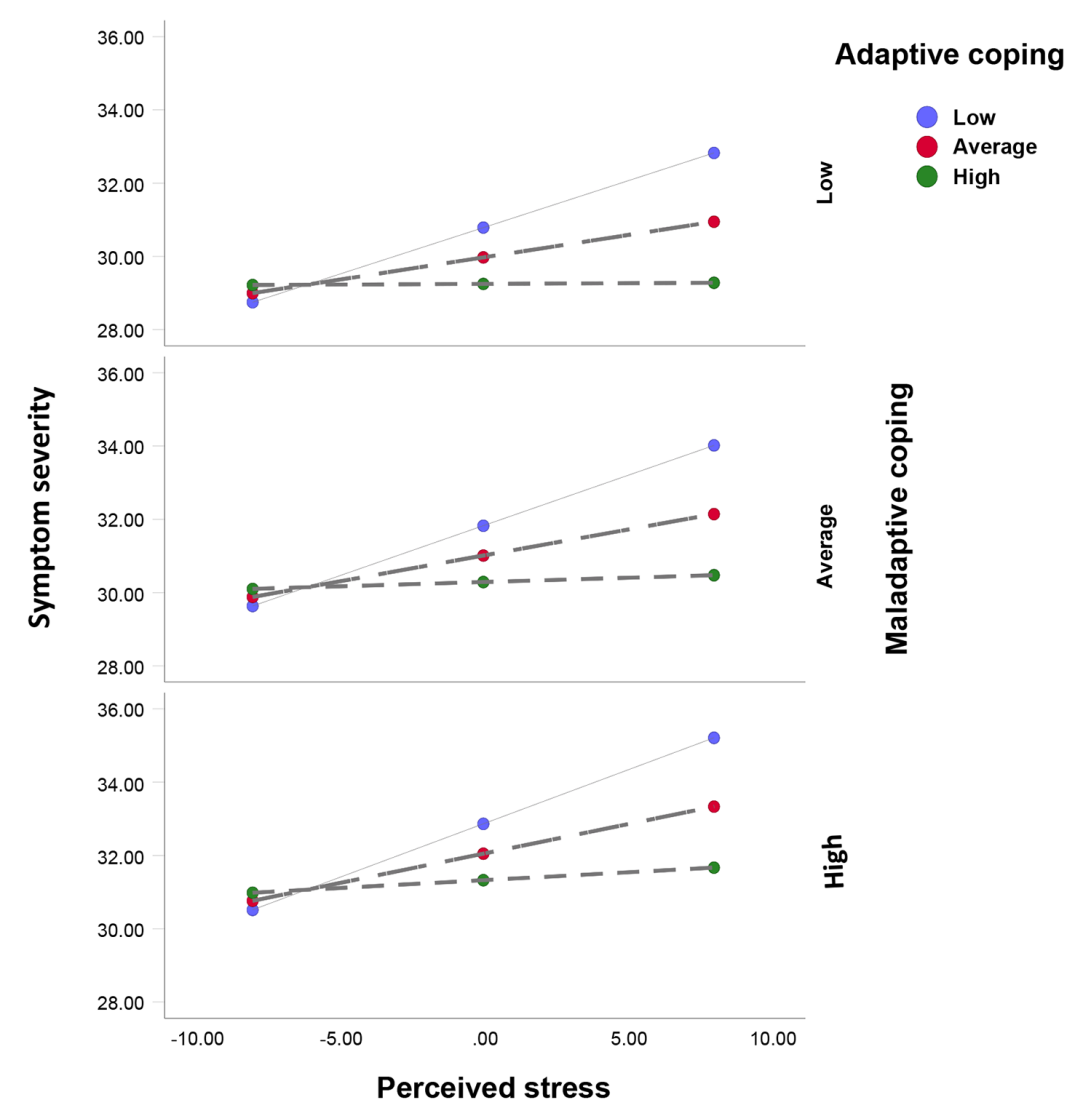
Conditional effect of perceived stress on symptoms severity at different levels of adaptive and maladaptive coping

**Fig. 4:**
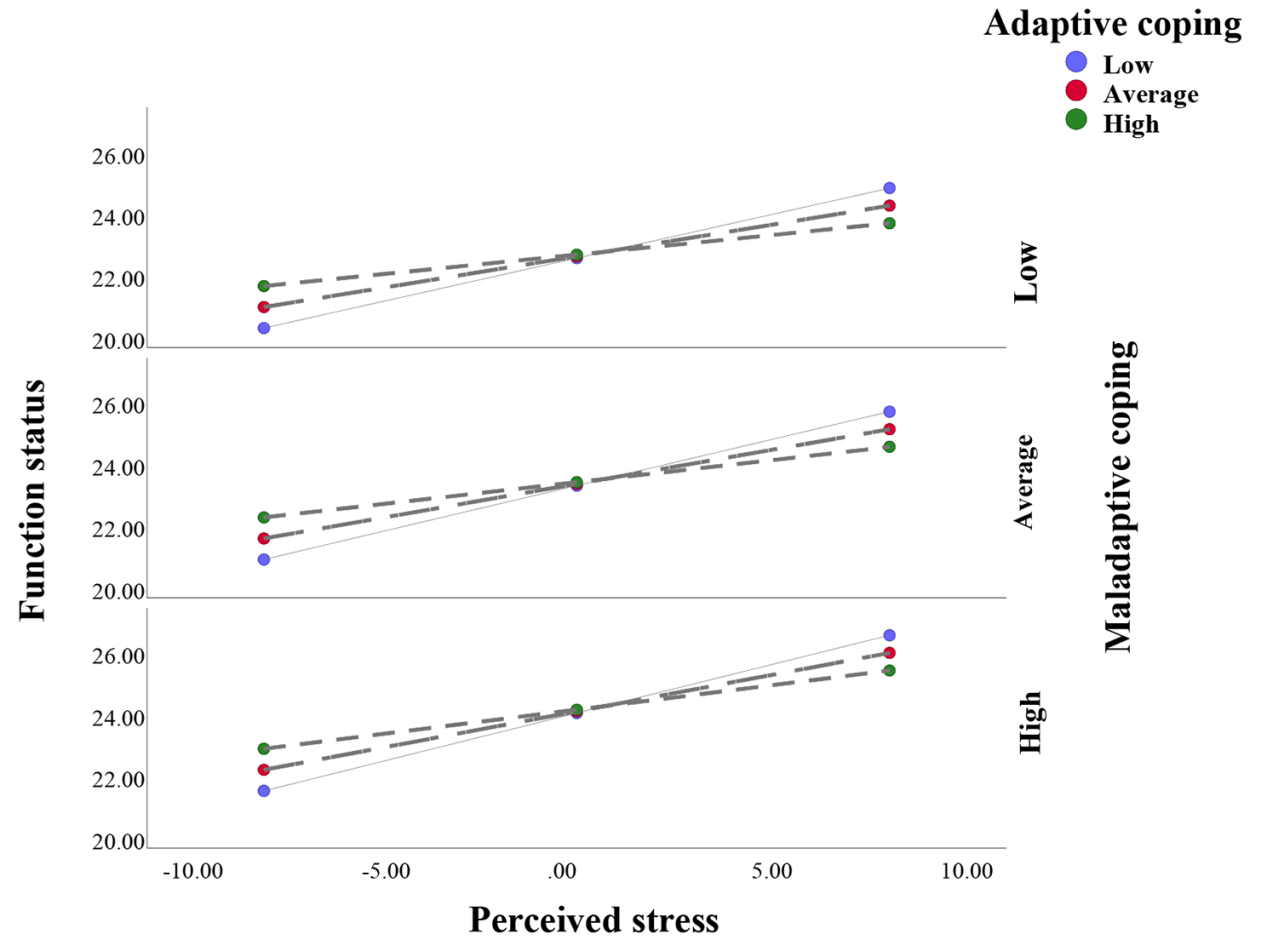
Conditional effect of perceived stress on function status at different levels of adaptive and maladaptive coping

The plots of the conditional effects of perceived stress on symptoms severity and functional status at different levels of adaptive and maladaptive coping strategies are illustrated in Figs. 3 and 4, respectively. The figures illustrate how coping strategies can moderate the relationship between perceived stress and the outcome variables. The figures show that the slopes of the regression lines vary depending on the levels of adaptive coping.

In Fig. 3, the effect of perceived stress on symptoms severity is positive and varies depending on the level of adaptive coping. When adaptive coping is low, the effect of perceived stress on symptoms severity is strong and positive, meaning that higher perceived stress leads to higher symptoms severity. When adaptive coping is high, the effect of perceived stress on symptoms severity is weak and positive, meaning that higher perceived stress leads to slightly higher symptoms severity. When adaptive coping is medium, the effect of perceived stress on symptoms severity is moderate and positive, meaning that higher perceived stress leads to moderately higher symptoms severity. The figure shows no effect of maladaptive coping on the relationship between perceived stress and symptoms severity because the interaction effect between perceived stress and maladaptive coping was not significant.

In Fig. 4, the slopes of the regression lines vary depending on the levels of adaptive and maladaptive coping. Specifically, as perceived stress increases, functional status increases for individuals with high adaptive coping, decreases for individuals with high maladaptive coping, and remains relatively stable for individuals with average or low levels of both types of coping. That suggests that adaptive coping can buffer the negative impact of perceived stress on functioning status, while maladaptive coping can exacerbate it.

## Discussion

The hallmark of contemporary nursing practice is a focus on providing patient-centered care based on empirical research findings. Carpal tunnel syndrome is a complex condition that has several physical and psychological repercussions that affect the quality of life of patients. Our study shed light on how controlling certain psychological factors, such as perceived stress and coping approaches, could affect the critical outcomes of CTS, such as functional status and severity of symptoms. In addition, our study aimed to explore the role of coping in the relationship between perceived stress and outcomes among patients with CTS. Our study proved that controlling stress and pursuing adaptive coping strategies among CTS patients could significantly improve their functional status while reducing the severity of symptoms.

### Perceived stress, symptom severity, and functional status of CTS patients

Our study found a positive and significant correlation between perceived stress and both symptom severity and functional status among CTS patients. This means that CTS patients with high perceived stress levels have devastating functional status and exhibit increased severity of symptoms. This is supported by the results of the regression analysis, which revealed a significant effect of perceived stress on symptoms severity and functional status among CTS patients. This also gives the impression that perceived stress was a powerful predictor of these outcomes, as every unit increase in perceived stress accounted for a reasonable change in symptoms severity and functional status (B = 0.148 and 0.218, respectively). Meanwhile, this finding is also reflected in the self-reporting of CTS patients in our study since there is a moderate level of perceived stress among them, parallel to a moderate level in both symptoms severity and functional status impairment.

These results can be explained in terms of the impact of stress on inflammation and swelling in the wrist, which can compress the median nerve and worsen CTS symptoms. Stress elicits the body’s “fight or flight” response, leading to the secretion of hormones (such as cortisol and adrenaline), which can cause inflammation and swelling. Inflammation in the wrist can exert pressure on the median nerve, resulting in symptoms like numbness, tingling, and weakness in the hand and wrist [1]. Another explanation for the relationship between stress and CTS is that stress can contribute to muscle tension and poor posture, which increase the risk of developing CTS. When individuals experience stress, they tend to hold their muscles in a tense and rigid position, leading to muscle fatigue and strain. Poor posture, such as slouching or hunching over a computer, can also place pressure on the wrist and compress the median nerve, thereby increasing the likelihood of CTS. Over time, chronic stress and poor posture can contribute to the development of CTS symptoms [47]. Our study supports this notion, as it reveals that 64.7% of participants are engaged in manual handwork, which is considered one of the risk factors for CTS.

It is important to note this relationship is expected, and it is the case in the studies of Jacob et al. (2023); Alendijani et al. (2023); Kurtul & Mazican (2023); and Jokar et al. (2022) [6, 8, 11, 48]. These studies found CTS patients with high anxiety and stress levels reported considerable improvement in their functional status, and their symptoms got worse with persistent stress, which is similar to our results. Moreover, our results are consistent with previous research that found that perceived stress is associated with increased pain intensity and disability in various chronic pain conditions, including low back pain [49], fibromyalgia [50], endometriosis [51], arthritis [52], migraine [53], multiple sclerosis [54], neuropathic pain, and a group of chronic overlapping pain conditions [55]. These conditions produce similar functional impairments, like CTS, which further supports our findings.

### Coping strategies, symptoms severity, and functional status among CTS patients

Our study revealed a negative association between adaptive coping (as planning, active coping, acceptance, positive reframing, emotional support, and humor) and perceived illness severity in terms of symptoms severity and functional status. While maladaptive coping strategies (such as denial, self-distraction, self-blame, and substance use) had a positive association, the adoption of these maladaptive coping strategies worsened the outcomes of CTS. In other words, effective adaptive coping of CTS patients is associated with decreased symptoms severity and improved functional status. This finding is evident in the results of the regression analysis in our study, which revealed a significant effect of coping on controlling the severity of symptoms and improving the functional status of CTS patients. This gives the impression that both adaptive and maladaptive coping strategies were significant predictors of symptoms severity, while maladaptive coping strategies were a significant predictor of functional status. This suggests that healthcare providers should assess patients’ coping strategies and provide interventions to promote adaptive coping strategies among patients with CTS.

This relationship could be explained by the fact that the adaptive coping of CTS patients with stress and disability produces significant relaxation, which is often associated with positive outcomes. Furthermore, the effect of adaptive coping on producing positive outcomes in CTS patients is expected and could be attributed to several reasons. Firstly, adaptive coping strategies such as splinting and ergonomic modifications can help alleviate pressure on the median nerve. By wearing a splint or making adjustments to their work environment, patients can reduce the strain on their hands and wrists, improving their ability to perform everyday tasks with less pain and discomfort. Secondly, adaptive coping may involve engaging in specific exercises and physical therapy techniques that can strengthen the muscles in the hand and wrist. This can help improve the stability and range of motion in these areas, leading to enhanced functional capability. By regularly practicing these exercises, patients can reduce the severity of their symptoms and enhance their overall functionality. Lastly, adaptive coping strategies can include lifestyle modifications, such as taking frequent breaks, stretching, and practicing relaxation techniques, to minimize the impact of repetitive motions and reduce stress levels, which further reduce pain intensity.

Our finding concerning this relationship is the case in the studies of Núñez-Cortés et al. (2023); Moro-López-Menchero et al. (2023); Daliri et al. (2022); Sun et al. (2019); Alsharif et al. (2022); and Kurtul & Mazican (2023) [4, 6, 10, 12, 18, 56]. There is a consensus in these studies that adaptive coping of patients with disease and the resultant stress could decrease pain perception among CTS patients, which further increases the pain threshold and decreases pain intensity among patients. In other words, these studies found adaptive coping could buffer the severity of symptoms of CTS and reduce them to a tolerable level that could enhance functionality, which is similar to our study. Moreover, the studies of Hudock et al. (2023); Jacob et al. (2023); and Omole et al. (2023) [1, 11, 20] revealed that CTS patients who adapt effectively through positive coping techniques reported high satisfaction and sickness presenteeism in their jobs, which also supports our finding and reflects that adaptive coping produces high tolerance for symptoms severity and enhances the functional milestones of daily life of CTS patients.

### The effect of coping on the linkage between perceived stress and both symptoms severity and functional status among CTS patients

Our study found that adaptive coping moderates the relationship between perceived stress and both symptoms severity and functional status among CTS patients, making it less positive. This means that higher levels of adaptive coping buffer the negative impact of perceived stress on CTS outcomes. However, maladaptive coping did not moderate these relationships. This gives the impression that adaptive coping is associated with less perceived stress and high positive outcomes in terms of decreased severity of symptoms and better functionality among CTS patients. This reinforces the importance of using adaptive coping techniques among CTS patients and makes it necessary for nurses to learn various adaptive coping strategies for better health education sessions for CTS patients.

It is obvious that the varying slopes of the lines at different levels of adaptive coping indicate that individuals with different coping strategies responded differently to perceived stress. In other words, when adaptive coping is low, there is a steep positive slope, indicating a strong and positive effect of perceived stress on the two outcomes. This suggests that for every one-unit increase in perceived stress, symptoms severity and function status increase significantly on average. Conversely, when adaptive coping is high, the slope is positive but flat, indicating that adaptive coping buffers the negative impact of perceived stress on symptoms severity and function status.

One possible explanation for these results is the effectiveness of adaptive coping strategies in attenuating the impact of perceived stress on symptoms severity. Individuals with high levels of adaptive coping may have developed effective strategies, such as problem-solving skills, seeking social support, or engaging in relaxation techniques, to manage stress. These individuals may possess greater stress resilience and can buffer the negative effects of stress on symptoms severity, resulting in a weaker relationship between perceived stress and symptoms severity [10, 12]. Conversely, individuals with low levels of adaptive coping may lack effective strategies to cope with stress. They may experience heightened emotional distress and struggle to manage their symptoms in the face of stress, which could explain the strong and positive relationship observed between perceived stress and symptoms severity [8, 57].

One important finding in our study is that the moderate slope observed for individuals with moderate levels of adaptive coping suggests that they possess some degree of stress management skills but may still be vulnerable to the negative impact of stress on symptoms severity. It is possible that their coping strategies are somewhat effective, but they may not eliminate the influence of stress on symptoms severity. Furthermore, the lack of a significant interaction effect between perceived stress and maladaptive coping suggests that maladaptive coping strategies may not have a substantial moderating effect on the relationship between perceived stress and symptoms severity. This could mean that maladaptive coping strategies, such as avoidance or self-destructive behaviors, do not effectively mitigate the impact of stress on symptoms severity in CTS.

Our results are the same as in the studies of Moro-López-Menchero et al. (2023); Dwedar et al. (2023); Sharief et al. (2019); Mansfield et al. (2018); and Rasid et al. (2016) [9, 10, 15, 58, 59]. These studies highlighted that individuals who used avoidance coping strategies were more likely to experience pain and disability associated with CTS. Additionally, individuals who rely on avoidance coping may be less inclined to seek medical attention for their symptoms, resulting in a delayed diagnosis and treatment of CTS. Also, these studies agree with our study on the importance of adaptive coping strategies in producing a positive quality of life among the affected persons with CTS. Another study conducted by Núñez-Cortés et al. (2022) concluded that it is important to evaluate stress, anxiety, and disease perception variables for CTS patients preoperatively, as this could help identify patients at risk of unfavorable surgical outcomes and enable timely treatment [16]. As further knowledge is gained regarding the role of cognitive and mental health factors and their potential impact on carpal tunnel release surgery, clinicians will be better equipped to effectively approach patients, which further supports our conclusions.

Overall, our results highlight the importance of adaptive coping strategies in influencing the relationship between perceived stress, symptoms severity, and function status in CTS. Developing and promoting effective coping skills may be beneficial in managing symptoms and improving functional outcomes in individuals with this condition.

### Strengths and limitations of the study

The study was conducted on a diverse sample of patients with CTS from three hospitals in Egypt, using valid and reliable instruments to measure the variables of interest, which enhanced the rigor of our study. The study controlled for potential confounding factors, such as demographic and biomedical data, in the analysis. The study adds to the existing knowledge about the psychological aspects of CTS by examining the moderating role of coping strategies in the relationship between perceived stress and both symptoms severity and function status.

The study has three limitations. First, this study used a cross-sectional design, which limits the causal inference between the study variables. Future studies should use a longitudinal design to establish causal relationships among them. Second, this study relied on self-reported measures, which may introduce biases such as social desirability or recall errors. Future studies should use objective measures, such as nerve conduction studies or functional tests, to assess symptoms severity and functional status. Third, this study used a convenience sample of patients from three hospitals in Egypt, which may limit the generalizability of the findings to other populations or settings. Future studies should use a larger and more representative sample of patients from different countries or regions.

### Implications of the study

#### Implications for research

Our study has several suggestions for future research. First, conduct longitudinal studies to examine the changes in perceived stress, coping strategies, functional status, and well-being over time in CTS patients and to identify the predictors and outcomes of these variables. Second, compare the effects of different psychological interventions on perceived stress, coping strategies, functional status, and well-being in CTS patients and determine the optimal type, duration, frequency, and intensity of these interventions. Third, explore the biological and physiological mechanisms that mediate the relationship between psychological factors and functional status in CTS patients and investigate the role of inflammation, oxidative stress, neuroplasticity, and epigenetics in this process. And finally, examine the generalizability of the findings to other nerve entrapment conditions and to other chronic pain conditions, and test whether psychological factors have a common or specific influence on disability across different conditions.

#### Implications for practice

Our findings have several implications for clinical practice. First, they suggest that nurses and patients should assess and address perceived stress and coping strategies in CTS patients, as they may affect their functional status and well-being. Second, they imply that interventions that aim to reduce stress and enhance adaptive coping may be beneficial for improving functional status in these patients. Third, they provide evidence for the complex interplay between psychological and physical factors in CTS and call for more studies to explore the underlying mechanisms and causal relationships among these variables. Fourth, they indicate that psychological factors may have a common influence on disability across different nerve entrapment conditions. Therefore, it may be useful to incorporate psychological assessment and intervention into the management of CTS and other similar conditions. Psychological interventions may include cognitive-behavioral therapy, mindfulness-based therapy, relaxation techniques, or psychoeducation. These interventions may help patients modify their negative thoughts and emotions about their condition, reduce their terrible pain, enhance their coping skills, improve their illness perceptions, and ultimately improve their outcomes.

## Conclusion

The study, involving 225 CTS patients, conducted two moderation analyses to explore how coping strategies influence the relationship between perceived stress and symptoms severity as well as functional status. Both models were significant, explaining over 70% of the variance in symptoms severity and functional status. The study results revealed that higher perceived stress was associated with worse outcomes, while adaptive coping was linked to lower symptoms severity. Maladaptive coping was associated with higher symptoms severity and functional status. In addition, perceived stress, adaptive coping, and maladaptive coping were significant predictors of symptoms severity and function status in CTS patients. Moreover, adaptive coping moderated the effects of perceived stress on both outcomes in a way that buffered the negative impact of perceived stress on CTS outcomes. This study contributes to the literature on the psychosocial aspects of CTS by highlighting the role of perceived stress and coping strategies in influencing the outcomes of CTS patients and suggesting potential avenues for interventions to improve patient well-being.

## Data Availability

The datasets used and/or analysed during the current study are available from the corresponding author on reasonable request.

## Abbreviations

CTS: Carpal Tunnel Syndrome
BMI: Body Mass Index
PSS: Perceived Stress Scale
BCTQ: Boston Carpal Tunnel Questionnaire
